# Impact of DsCPV-1 Passage Through Alternative Host *Manduca sexta* on Its Virulence, Productivity, and Transmission Potential in Lepidopteran Forest Pests

**DOI:** 10.3390/insects16121217

**Published:** 2025-11-28

**Authors:** Anna O. Subbotina, Yuriy B. Akhanaev, Elena L. Arzhanova, Irina A. Belousova, Alexey V. Kolosov, Boris S. Kondrashov, Sofia S. Melnikova, Vyacheslav V. Martemyanov

**Affiliations:** 1 Scientific Center of Genetics and Life Sciences, Sirius University of Science and Technology, Olympic Avenue 1, Sirius 354340, Russia; subbotinaanya11@gmail.com (A.O.S.); akhanaev@mail.ru (Y.B.A.); elena.arzhanova@hotmail.com (E.L.A.); belousova_i@yahoo.com (I.A.B.); 2 Institute of Systematics and Ecology of Animals, Siberian Branch of Russian Academy of Sciences, Novosibirsk 630091, Russia; 3 FBRI State Research Center of Virology and Biotechnology VECTOR, Rospotrebnadzor, Koltsovo 630559, Russia; kolosov@vector.nsc.ru; 4 Department of Natural Science, Novosibirsk State University, Pirogova Str. 1, Novosibirsk 630090, Russia; boris.kondrashov.biology@gmail.com (B.S.K.); melnikova18072005@gmail.com (S.S.M.)

**Keywords:** *Dendrolimus sibiricus*, *Lymantria dispar*, *Manduca sexta*, *Cypovirus*, polyhedra, passage

## Abstract

*Lymantria dispar* (L.) and *Dendrolimus sibiricus* Tschetv. are lepidopteran forest pest species, with a long history of outbreak dynamics. The recently isolated strain of Cypovirus—Dendrolimus sibiricus cypovirus-1 (DsCPV-1) shows potential as a bioinsecticide against these and other lepidopteran species. *Manduca sexta* (L.) has been identified as a promising producer of DsCPV-1. Although *M. sexta* offers clear advantages as an alternative host for DsCPV-1 production, the DsCPV-1 isolate passaged through *M. sexta* (DsCPV-Ms) produces fewer polyhedra than the original isolate. Here, we evaluated the virulence, recovery of polyhedron formation, and replication of the DsCPV-Ms in *L. dispar* (alternative host) and *D. sibiricus* (original host) larvae to assess its suitability as a biocontrol agent in these hosts. Our results demonstrate that DsCPV-Ms causes significant mortality along with efficient polyhedra synthesis in *D. sibiricus* larvae. In contrast, DsCPV-Ms infection of *L. dispar* resulted in no significant mortality despite detectable viral replication and polyhedron formation. Polyhedron formation in *L. dispar* was significantly lower following infection with DsCPV-Ms than with the original isolate, despite confirmed replication of DsCPV-Ms. These findings indicate that DsCPV-Ms remains effective against *D. sibiricus*; however, further improvements are needed before it can be applied to *L. dispar*.

## Simple Summary

This study investigated the biological efficacy of DsCPV-1, a cypovirus strain originally isolated from *Dendrolimus sibiricus* Tschetv., after passage through the alternative, promising producer host *Manduca sexta* (L.), to evaluate its potential as a biocontrol agent against two lepidopteran forest pests. Virulence of DsCPV-1 passaged through *M. sexta* was assessed in the original host (*D. sibiricus*) and the alternative host (*Lymantria dispar*) and compared with that of DsCPV-1 passaged through its original host. Polyhedron productivity of DsCPV-1 passaged through *M. sexta* was examined in *D. sibiricus* and *L. dispar* and compared with productivity observed when the DsCPV-1 was passaged in *D. sibiricus* and *M. sexta* to determine whether polyhedron formation, and thus transmission potential, is restored. Exposure to DsCPV-1 passaged through *M. sexta* caused high mortality and polyhedron formation in *D. sibiricus*, confirming its promise as a biocontrol agent for this pest. In contrast, DsCPV-1 passaged through either *D. sibiricus* or *M. sexta* induced no significant mortality in *L. dispar*, despite viral replication and polyhedron formation, suggesting mid-instar larvae are tolerant to infection. Polyhedron production by DsCPV-1 passaged through *M. sexta* was restored in *D. sibiricus* but not in *L. dispar*.

## 1. Introduction

*Lymantria dispar* (L.) (Lepidoptera: Erebidae), a polyphagous defoliator [1], is listed among the world’s top 100 invasive species (“GISD,” n.d.) (accessed on 18 June 2025) and causes extensive damage to broadleaf forests [2]. *Dendrolimus sibiricus* Tschetv. (Lepidoptera: Lasiocampidae) is a conifer pest that leads to large-scale forest damage during population outbreaks across Northern Asia [3] and has been identified as a potential emerging invasive pest species, raising concerns about its possible establishment in new regions [4,5,6].

In response to increasing ecological concerns over chemical pesticides, there is growing interest in developing biological control agents that are both effective and environmentally friendly. A recently identified cypovirus strain, Dendrolimus sibiricus Cypovirus-1 (DsCPV-1), has shown promising potential as a biocontrol agent against several lepidopteran pest species, including forest and agricultural pests [7], as well as demonstrating biological safety for non-target invertebrates [8]. This RNA virus, originally isolated from *D. sibiricus* larvae, belongs to the family *Spinareoviridae*, genus Cypovirus (ICTV) which is characterized by the production of occlusion bodies (OBs) known as polyhedra [9]. Polyhedra-forming viruses encapsulate infectious viral particles into OBs and favor their persistence in the environment, especially in soil, until they are consumed by a susceptible insect, remaining infective for long periods during low host population phases (i.e., diapause, seasonal reasons, etc.) [10,11,12].

To facilitate large-scale production of DsCPV-1, an efficient and cost-effective production method is required. *Manduca sexta* (L.) (Lepidoptera: Sphingidae), one of DsCPV-1’s alternative hosts, is a promising species for in vivo DsCPV-1 production due to its rapid development, low rearing costs during routine farming, ability to support DsCPV-1 replication, and capacity to cause mortality in initial host *D. sibiricus* larvae [7,13]. However, the ability of the isolate obtained from *M. sexta* (hereafter referred to as DsCPV-Ms isolate) to cause mortality in other target lepidopteran species has not been demonstrated and the mortality rate has not been characterized in any study yet. In addition, previous studies have shown that DsCPV-1 passaged through *M. sexta* exhibits atypical pathogenesis, which is characterized by reduced polyhedra formation when compared to the original isolate obtained from *D. sibiricus* (DsCPV-Ds isolate) [13]. Although the reduced number of polyhedra in DsCPV-Ms isolates may potentially be compensated by polyhedra formation in infected larvae treated with this suspension, the ability of lepidopteran larvae to produce polyhedra following DsCPV-Ms infection has not yet been investigated.

Therefore, to assess the potential effectiveness of a bioinsecticide based on DsCPV-1 passaged through the alternative host *M. sexta*, this study was conducted to evaluate the virulence and polyhedra production of this viral isolate in alternative host larvae against two economically significant forest pest species.

## 2. Materials and Methods

### 2.1. Insects and DsCPV-1

Fourth-instar larvae of *D. sibiricus* were collected in the forest-steppe zone of southern Western Siberia (52.039° N, 80.3090° E), Russia. The larvae were reared at +24 °C and 40% to 60% relative humidity under the 16:8 light:dark regime and were fed the shoots of the larch *Larix sibirica*. 

Egg masses of diapausing *L. dispar* were collected in the Novosibirsk region, Western Siberia (54.2342° N, 82.7614° E), Russia. The eggs were stored at +4 °C throughout the winter diapause. Larval hatching was synchronized with birch leaf budburst (occurring in the first third of May), according to Martemyanov et al., 2015 [14]. After hatching, the larvae were reared on cut branches of silver birch (*Betula pendula* Roth) under controlled conditions of +22 °C temperature, natural humidity, and a natural photoperiod.

Polyhedra of DsCPV-Ds were isolated from cadavers of *D. sibiricus* larvae initially infected by DsCPV-1 isolate according to Martemyanov et al., 2023 [7]. The DsCPV-Ms suspension was prepared from 20 dead *M. sexta* larvae infected with the DsCPV-Ds isolate. After removing the cuticle, the internal tissues of the larvae were homogenized, diluted with water, and filtered through a series of gauze to produce a clarified suspension. Final suspension of DsCPV-Ms isolate was 22 mL.

### 2.2. Challenging D. sibiricus and L. dispar Larvae with DsCPV-1 Isolates

Fifth-instar larvae of *D. sibiricus* were used for infection with suspension of DsCPV-1-Ms isolate. This suspension was diluted 20-, 200-, 2000- and 20,000-fold, and a volume 10 mL of each dilution was applied by spraying onto branches of larch, dried and were later consumed by *D. sibiricus* larvae. Mortality was recorded daily for 8 days in both the infected and control groups. Each infected group consisted of three replicates, with 15 larvae per replicate, for a total of 45 larvae per dilution (20-fold, 200-fold, 2000-fold, and 20,000-fold). Similarly, the control group comprised three replicates of 15 untreated larvae each (*n* = 45). DsCPV-1 infection data for *D. sibiricus* were obtained from Martemyanov et al., 2023 [7].

Third-instar larvae of *L. dispar* were used for infection with different isolates of DsCPV-1. For DsCPV-Ds suspension, concentration was 5 × 10^7^ polyhedra/mL. We also used a 20-fold dilution of DsCPV-1-Ms isolate to infect *L. dispar* larvae, as its virulence in *D. sibiricus* larvae was shown to be equivalent to that of a 5 × 10^7^ polyhedra/mL (Martemyanov et al., 2023 [7] and this study). Both suspensions, with a volume 10 mL, were applied by spraying onto branches of *B. pendula*, which were dried and later consumed by *L. dispar* larvae. Mortality was recorded daily for 14 days in both the infected and control groups. Each group consisted of five replicates with 10 larvae per replicate, resulting in a total of 50 larvae per group (*n* = 50).

Dead larvae of *D. sibiricus* infected with the DsCPV-Ms isolate and of *L. dispar* infected with both the DsCPV-Ds and DsCPV-Ms isolates were individually homogenized using a polyurethane pestle. The polyhedra productivity of the DsCPV-1 isolates was estimated with light microscopy (400×) using a hemocytometer.

### 2.3. qPCR Assay

qPCR was applied to confirm DsCPV-1 replication in *L. dispar* larvae infected with DsCPV-Ds and DsCPV-Ms. For this analysis, five randomly selected infected larvae were dissected and their gut tissues collected on day 1, and an additional set of five asymptomatic infected larvae were sampled on day 14, from both isolates. These were not the same larvae as those in the mortality group, as we maintained a separate larval group to prevent sample depletion due to sampling. Total RNA was extracted using the Lira-reagent (BioLabMix, Novosibirsk, Russia), followed by reverse transcription using the RT-M-MuLV-RH kit (BioLabMix, Novosibirsk, Russia) and qPCR using the BioMaster HS-qPCR SYBR Blue kit (BioLabMix, Novosibirsk, Russia), according to Subbotina et al., 2025 [13]. The primers used for amplification of the DsCPV-1 polyhedrin gene were designed as follows (5′–3′): forward primer, TCTCACCGAATGCTTACCCA; reverse primer, AGAGCGTCACCCTATCCGAA. To enable accurate comparison of Cq values, the extracted RNA samples were diluted identically, according to Belevich et al., 2024 [8].

To determine the viral load in the DsCPV-Ms isolate, a 50 μL aliquot of a 20-fold dilution of the DsCPV-Ms suspension was processed for RNA isolation. RNA extraction, reverse transcription, and amplification were performed using the same protocols as those applied to larval intestinal samples. For the standard curve, a pAL2-T plasmid vector containing a fragment of the polyhedrin gene (GenBank: OL774561.1) was constructed. The 153 bp fragment (sequence: 5′-TCTCACCGAATGCTTACCCATACCTCGACATCAATAACCATAGCTATGGAGTAGCTCTGAGTAACCATCAGTGATTGCTCGTGTAACTTGGATACCAGAAAACATGACGCCGTGATGAATTACGCGCCCGGTCTTCGGATAGGGTGACGCTCT-3′) was amplified using the same primers and qPCR protocol described in Subbotina et al., 2025 [13], and subsequently cloned into the pAL2-T vector using the Quick-TA Cloning Kit (Evrogen, Russia) according to the manufacturer’s protocol. The ligation product was chemically transformed into *XL1-Blue* competent cells (Evrogen, Russia), and transformants were cultured for 12 h at 37 °C. Plasmid DNA was isolated with the Plasmid-10-Mini Kit (Biolabmix, Russia) in accordance with the kit protocol. A standard curve was generated using serial 2-fold dilutions of the pAL2-T plasmid vector containing a polyhedrin gene insert, with a known copy number per PCR reaction: DNA (copy) = (6.02 × 10^23^(copy/mol) × DNA concentration(g/μL))/(M_vector with insert_(g/mol)) [15]. The copy number per mL of the original suspension was then calculated by accounting for sample dilution factors and volume of aliquot of suspension.

### 2.4. Statistical Analysis

Mortality in groups of *D. sibiricus* and *L. dispar* larvae, polyhedra productivity and qPCR data in groups of *L. dispar* larvae were tested using a Mann–Whitney U test (Statistica 6.0). Polyhedra productivity in *D. sibiricus* larva was tested using Kruskal–Wallis test followed by Dunn’s post hoc test (in PAST 3). Data were preliminarily checked for outliers.

## 3. Results

**Figure 1:**
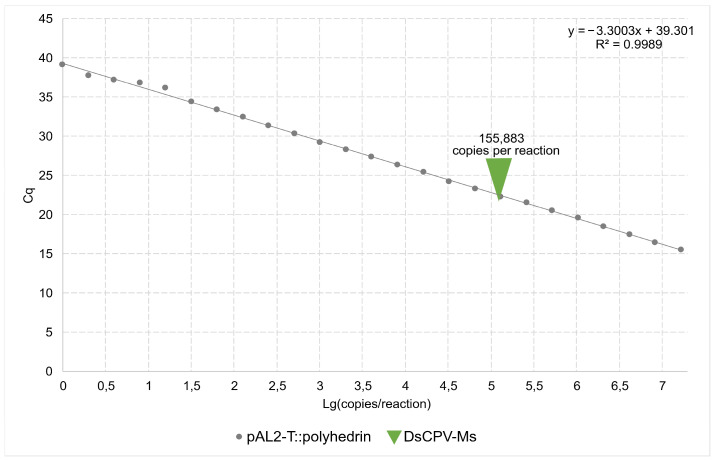
Quantitative PCR for the pAL2-T plasmid vector carrying a polyhedrin gene insert and DsCPV-Ms suspension samples.

The viral load of the DsCPV-Ms isolate was quantified by qPCR using a standard calibration curve (y = −3.3003x + 39.301; Figure 1). The copy number in the DsCPV-Ms suspension sample was 155,883 copies per PCR reaction. After accounting for dilution factors and sample volume, the viral titer in the DsCPV-Ms suspension was calculated as 3.0 × 10^8^ copies/mL.

**Figure 2:**
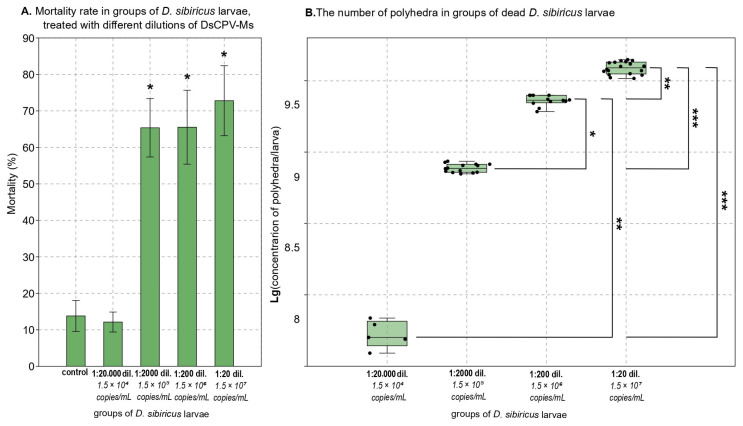
(**A**): * *p* < 0.05 versus the control (Mann–Whitney U test). (**B**): Brackets indicate pairs of groups with statistically significant differences (Dunn’s post hoc test); asterisks denote significance levels: * *p* < 0.05, ** *p* < 0.01, *** *p* < 0.001.

Subsequently, 20-fold, 200-fold, 2000-fold, and 20,000-fold dilutions of this DsCPV-Ms suspension were used to infect *D. sibiricus*, and the 20-fold dilution was used to infect *L. dispar*. The corresponding viral concentrations were as follows: 20-fold dilution—1.5 × 10^7^ copies/mL, 200-fold—1.5 × 10^6^ copies/mL, 2000-fold—1.5 × 10^5^ copies/mL, and 20,000-fold—1.5 × 10^4^ copies/mL (Figure 2A,B).

Exposure to the 20-fold dilution of DsCPV-Ms isolate resulted in 72.8% mortality (control vs. infected: Z = −1.964, *p* = 0.049; Mann–Whitney U test); 65.6% at the 200-fold dilution (control vs. infected: Z = −1.964, *p* = 0.049), 65.4% at the 2000-fold dilution (control vs. infected: Z = −1.964, *p* = 0.049) and 12.1% at the 20,000-fold dilution in *D. sibiricus* larvae (control vs. infected: Z = 0.00, *p* = 1.00; Mann–Whitney U test; Figure 2A).

In dead DsCPV-Ms infected *D. sibiricus* larvae, polyhedra were detected by light microscopy. For 20-fold dilution of DsCPV-Ms isolate, the median productivity in five-instar larvae was 3.9 × 10^9^ polyhedra/larvae with a range of 3.3 × 10^9^ to 4.4 × 10^9^; for 200-fold dilution—2.3 × 10^9^ polyhedra/larvae with a range of 1.9 × 10^9^ to 2.5 × 10^9^; for 2000-fold dilution—7.7 × 10^8^ polyhedra/larvae with a range of 7.0 × 10^8^ to 8.6 × 10^8^; for 20,000-fold dilution—5.0 × 10^7^ polyhedra/larvae with a range of 3.9 × 10^7^ to 6.9 × 10^7^ (Kruskal–Wallis test: H_(3, N=49)_ = 43.79008, *p* < 0.001; see results of Dunn’s post hoc test on Figure 2B).

**Figure 3:**
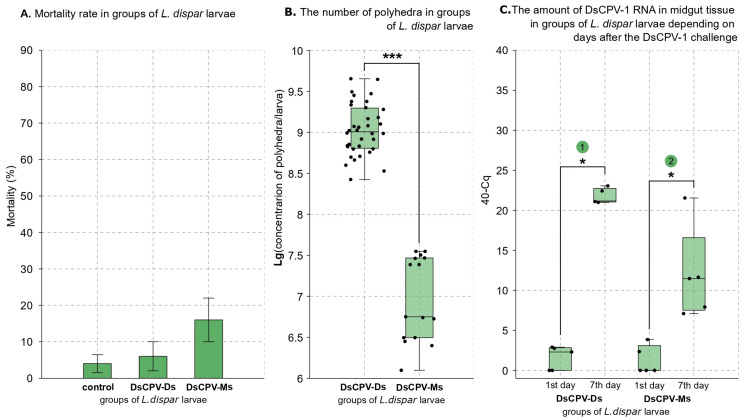
(**A**): * *p* < 0.05 versus the control (Mann–Whitney U test). (**B**): Brackets indicate comparable groups; asterisks denote significant differences between those groups: *** *p* < 0.001 (Mann–Whitney U test). (**C**): Brackets indicate comparable groups; asterisks denote significant differences between those groups: * *p* < 0.05 (Mann–Whitney U test); 1. *p* = 0.012, 2. *p* = 0.011 (Mann-Whitney U test).

To infect *L. dispar* larvae, we used a concentration of 5 × 10^7^ polyhedra per milliliter for DsCPV-Ds suspension and a 20-fold dilution of stock DsCPV-Ms. These concentrations were selected because they demonstrated comparable virulence effects in studies: against *L. dispar* (Martemyanov et al., 2023 [7]) and *D. sibiricus* (this study). No significant differences in mortality were observed between the control and infected larvae in this experiment (control vs. infected of DsCPV-Ds: Z = −0.209, *p* = 0.811; control vs. infected of DsCPV-Ms: Z = −1.462, *p* = 0.118; Mann–Whitney U test; Figure 3A).

In *L. dispar* larvae infected with both viral isolates, polyhedra were successfully detected by light microscopy. The proportion of samples in which polyhedra were observed was higher in larvae infected with the DsCPV-Ds isolate (90.7%) compared to those infected with the DsCPV-Ms isolate (41.0%). The median productivity of polyhedra during infection significantly differs between the two isolates. For the DsCPV-Ds isolate, the median yield was 1 × 10^9^ polyhedra/larva, with a range of 2.7 × 10^8^ to 4.5 × 10^9^, while DsCPV-Ms isolate exhibited a lower median productivity of 5.6 × 10^6^ polyhedra/larva, with a range of 1.2 × 10^6^ to 3.5 × 10^7^ polyhedra/larva (Z = 5.581942, *p* < 0.001; Mann–Whitney U test; Figure 3B).

To confirm DsCPV-1 replication in *L. dispar* larvae infected with DsCPV-Ds and DsCPV-Ms isolates, qPCR was performed, as no significant mortality was observed in the DsCPV-Ds treated group, and neither significant mortality nor polyhedron formation was detected in most individuals from the DsCPV-Ms-treated group. Using the comparative Cq method with equally diluted samples [8], we demonstrated that the amount of DsCPV-1 dsRNA significantly increased over the infection in larvae infected with DsCPV-Ds isolate compared to the control group (Z = 2.5143, *p* = 0.012; Mann–Whitney U test; Figure 3C), as well as in those infected with DsCPV-Ms isolate compared to the control group (Z = 2.5377, *p* = 0.011; Mann–Whitney U test; Figure 3C).

## 4. Discussion

Our results demonstrate high mortality in fifth-instar larvae of *D. sibiricus* (72.8%) following infection with the 20-fold dilution of DsCPV-1 passaged through *M. sexta* (i.e., DsCPV-Ms), confirming its strong virulence and potential as a biocontrol agent against *D. sibiricus*. This is particularly advantageous, as rearing *D. sibiricus* for virus production is constrained by its prolonged life cycle, lasting two to five years [16], and its strictly seasonal availability, whereas *M. sexta* can be cultured (or even farmed) year-round under standard conditions and supported reliable viral amplification. In addition, based on mortality data from *D. sibiricus* infections with the DsCPV-Ms isolate and our previous studies, we selected a 20-fold dilution of the DsCPV-Ms as the pathogenic equivalent of 5 × 10^7^ polyhedra/mL of the DsCPV-Ds for infecting *L. dispar*. We selected treatment concentrations based on their biological effect, not the number of virus particles. This way, we compared polyhedron production in *L. dispar* under equally pathogenic conditions, an approach that better approximates conditions expected in future field applications. 

In our previous study, we showed that polyhedron productivity in middle-instar *D. sibiricus* larvae infected with original DsCPV-1 suspension at a concentration of 2 × 10^7^ polyhedra/mL averaged 1.0 × 10^8^ polyhedra per larva, with a range of 2.5 × 10^7^ to 3.1 × 10^8^ [7]. In the current study, *D. sibiricus* larvae infected with DsCPV-Ms isolate exhibited high levels of polyhedron formation across all treatment concentrations (medians ranging from 3.9 × 10^7^ to 5.0 × 10^9^ polyhedra per larva), which were comparable to or exceeded polyhedron productivity observed in the previous DsCPV-Ds infection. The higher yields may be attributed to the use of later-instar larvae in the present study, as larger larvae tend to produce more polyhedra [17,18]. Nevertheless, these results suggest that polyhedron formation is restored in the original host *D. sibiricus* infected with the DsCPV-Ms isolate and reaches levels comparable to those observed in *D. sibiricus* larvae infected with original DsCPV-1. A similar phenomenon—restoration of polyhedron formation upon reverse passage through the insect host—is observed in baculoviruses, which exhibit a “passage effect” characterized by reduced polyhedron production and infectivity following prolonged propagation in cell culture [19,20,21,22]. Despite their phylogenetic divergence, baculoviruses and cypoviruses have functional and evolutional parallels in polyhedron formation [23]. Thus, shifts in dominant phenotypes induced by intrahost bottlenecks could underlie polyhedron loss and recovery in both groups [24,25]. Although the specific mechanisms driving the emergence of polyhedron-free phenotypes in the DsCPV-Ms isolate (genetic or host-mediated) remain unknown. In the context of future application of the DsCPV-Ms as a biological control agent, the restoration of high-level polyhedron production will allow for the persistence of a large number of viral particles in the environment, thereby reducing the concentration needed for subsequent treatments.

Although previous studies demonstrated substantial mortality rates in second-instar *L. dispar* larvae (72.5%) following exposure to 10^7^ polyhedra/mL of the DsCPV-1 [7] and 33.5% mortality following exposure to 10^6^ polyhedra/mL of DsCPV-1 [26], as well as 72.8% mortality in late-instar *D. sibiricus* larvae after DsCPV-Ms treatment (current study), the present study failed to detect any significant differences in mortality among *L. dispar* groups treated with either viral passage compared to the control group. Despite this viral replication occurs in both infected groups, as evidenced by both microscopy screening of polyhedra and qPCR analysis. The absence of significant mortality despite confirmed infection in experimental *L. dispar* larvae suggests the presence of tolerance, defined as the ability to mitigate the negative effects of a pathogen on host fitness without reducing pathogen load, rather than resistance, which involves suppressing pathogen replication or accumulation [27,28,29]. 

No significant mortality was observed in *L. dispar* larvae infected with DsCPV-Ds isolate compared to the control group. Given that the same viral suspension caused high mortality in younger larvae in our previous study [7], the current results could indicate an age-related reduction in sensitivity. A similar instar-dependent effect has been demonstrated in *Spodoptera exigua* larvae infected with a Cypovirus [30]. Numerous studies have documented age-associated increases in non-specific immune parameters, both basally [31,32] and when stimulated by various pathogens [31,33], specifically in Lepidoptera [34,35,36] and in *L. dispar* [37,38,39]. Since the primary target tissue of Cypovirus is the midgut [9], enhanced intestinal defense mechanisms may contribute to tolerance of *L. dispar* larvae to DsCPV-1 infection [40,41]. One plausible mechanism involves the exfoliation of midgut epithelium during molting processes [36,42] and through cell proliferation [43,44,45], which could potentially provide healthy cells for organismal survival. However, this hypothesis requires further experimental validation. This could be addressed by implementing earlier treatments to target the most sensitive developmental stages, along with the addition of biologically active compounds such as optical brighteners, which have demonstrated their effectiveness in reducing lethal doses of Cypovirus in *L. dispar* [7] and other viruses in lepidopteran insects [30,46,47].

Polyhedron formation was observed in groups of *L. dispar* larvae infected with both isolates. While the extent of polyhedron formation varied significantly across isolates, the use of biological effect-based normalization, rather than quantification relative to infectious particle input, precludes definitive conclusions regarding whether these differences in polyhedron formation arise from variations in initial viral entry or post-entry replication dynamics. Notably, a substantial proportion (59%) of individuals infected with the DsCPV-Ms isolate failed to produce detectable polyhedra in *L. dispar*, despite positive qPCR signals confirming viral replication. This phenotype closely resembles the infection pattern previously reported in *M. sexta* [13]. While we observed polyhedron restoration in the original host, *D. sibiricus*, likely due to selection pressure favoring polyhedron-forming variants, as they are more readily transmitted within the original host population [24,25], this pressure apparently does not occur in the alternative host, *L. dispar*.

In conclusion, our results demonstrate that DsCPV-1 passaged through *M. sexta* retains high virulence and efficient polyhedron production, which preserves its transmission potential in *D. sibiricus* populations. These findings highlight DsCPV-Ms isolate of DsCPV-1 promise as a biologically effective agent for the control of *D. sibiricus*. In contrast, although DsCPV-1 replicates in *L. dispar*, neither DsCPV-Ds nor DsCPV-Ms infection resulted in significant mortality of middle instar larvae, a pattern that may reflect age-related insensitivity. Furthermore, reduced polyhedra formation following DsCPV-Ms indicates a failure to restore occlusion body production after passage through *M. sexta*. Thus, although DsCPV-1 passaged through *M. sexta* retains high virulence and efficient polyhedron production in its original host, *D. sibiricus*, its application against alternative hosts such as *L. dispar* requires further optimization.

## Data Availability

Dataset available on request from the authors.
